# Bridging the trust gap: the mediating role of patient satisfaction in physician empathy

**DOI:** 10.3389/fmed.2025.1647105

**Published:** 2025-09-04

**Authors:** Huanxi Bai, Fei Li, Zhifang He

**Affiliations:** School of Humanities, Jiangxi University of Traditional Chinese Medicine, Nanchang, China

**Keywords:** physician empathy, patient satisfaction, patient trust, doctor-patient relationship, medical environment

## Abstract

**Introduction:**

To explore the mediating effect of patient-perceived physician empathy on patient trust and patient satisfaction.

**Methods:**

The data of 834 outpatients in a top-three hospital in Jiangxi province were collected, and the general data questionnaire, diagnosis and treatment relationship empathy scale, patient trust scale and PDRQ-15 scale were used to investigate the relationship between variables, and the mediation effect was analyzed by process.

**Results:**

There was a significant positive correlation between doctors' empathy and patients' trust. Patient satisfaction plays a partial mediating role between perceived physician empathy and patient trust. Physician empathy affects patient trust partly through patient satisfaction.

**Discussion:**

On the basis of ensuring the quality of outpatient treatment, hospitals should strive to improve the doctor's visiting ability and service quality, so as to improve the patient's medical experience and satisfaction level, and thus ease the doctor-patient relationship.

## 1 Introduction

The recurrent nature of medical incidents imposes a substantial psychological burden on medical professionals and exerts undue professional pressure on them. The occurrence of medical incidents has been demonstrated to further erode this crucial trust (1). According to the “White Paper on the Practice Status of Doctors in China”, released in January 2018, 62% of doctors in China have encountered medical disputes of varying degrees, and 66% of doctors have experienced some form of conflict with patients. A substantial body of research has been conducted by scholars, aiming to explore the underlying causes of doctor-patient conflicts from diverse perspectives. Notable among these are the uneven distribution of medical resources, high patient expectations, asymmetry of medical information, and shortcomings of the medical system. Addressing these challenges necessitates a collaborative effort among government entities, healthcare organizations, healthcare professionals, and patients (2).

The significance of physician empathy in the doctor-patient relationship is well-documented. Patients have the expectation that physicians will be able to understand their pain and condition, yet many public hospital physicians fail to meet this expectation (3).The ability of physicians to communicate effectively with their patients has been demonstrated to foster an increase in patient trust (3).

The notion of patient satisfaction emerged within the domain of marketing as a derivative of customer satisfaction theory. Pascore (1983), a pioneering scholar in the field of patient satisfaction, posited that this concept encompasses individuals' sentiments and encounters concerning their fundamental needs, including health, illness, and quality of life. It also encompasses their subjective assessments of healthcare services received during the course of seeking medical care (4). Furthermore, patient satisfaction has been demonstrated to exert a significant influence on patient trust. The correlation between heightened patient satisfaction and elevated patient trust is well-documented (3).

Patient trust is defined as the belief that physicians possess the necessary skills to diagnose and treat illnesses, while also prioritizing the patient's interests, with numerous studies indicating that patient trust can lead to positive outcomes, including enhanced communication, improved patient satisfaction, and a stronger physician-patient bond (5). The level of medical technology and the attitude of medical personnel have a more significant impact on patients' experience of medical treatment, the doctor-patient relationship, and trust between doctors and patients (6). Existing research on patient trust has focused on patients' interpersonal trust in physicians (7). However, globally, patients' trust in physicians has been on a downward trend, and patient trust is affected by a variety of factors such as social background, medical conditions, and individual patient and physician characteristics (7).

Empathy in physicians has been demonstrated to be an effective strategy for enhancing patient satisfaction (8). Current research indicates Empathy enables physicians to communicate more effectively with patients, leading to a reduction in misunderstandings and misinformation. Consequently, patients' trust in their physicians is increased. Empathy has also been demonstrated to alleviate patients' anxiety and fear, thereby increasing their willingness to collaborate with their physicians, which in turn fosters a more robust physician-patient trust relationship (9). Empathy has been shown to enhance patients' trust in medical staff, which, in turn, has been demonstrated to positively impact patient satisfaction, treatment adherence, and treatment outcomes (10).

Current research indicates the level of satisfaction directly correlates with the degree to which patients recognize the quality of medical services, which is of significant importance for enhancing the doctor-patient relationship (2, 11). Empathic physicians have been shown to engender a sense of understanding and respect in patients, thereby enhancing their overall satisfaction with healthcare services and fostering greater trust in physicians. Research has demonstrated that patient satisfaction is a significant predictor of the satisfaction of the doctor-patient relationship across various levels of hospitals, and low patient satisfaction with hospital services can significantly exacerbate the tension in the doctor-patient relationship. In contrast, when patients are satisfied with healthcare services, they are more likely to trust their doctors and adhere to their medical prescriptions for treatment (12).

Despite the evident interrelationships among these three factors, previous studies have frequently overlooked their mutual relationships. The majority of research in this field has focused on examining the direct effects of doctor empathy on either patient trust or patient satisfaction, separately, without considering the mediating roles that one factor may play in the relationship between the other two. The objective of this research is to address this lacuna by conducting a comprehensive investigation into the interrelationship among doctor empathy, patient satisfaction, and patient trust. The study will provide novel insights into the doctor-patient relationship and offer practical implications for enhancing the quality of healthcare services. The objective of this initiative is to facilitate a more comprehensive comprehension of the doctor-patient relationship, whilst concurrently providing healthcare providers with pragmatic methodologies to disrupt the pernicious cycle and thereby engender a more favorable and efficacious healthcare milieu.

## 2 Materials and methods

### 2.1. Participants

This study is a cross-sectional descriptive study. This study was conducted at a tertiary hospital in Jiangxi Province. The selection of this hospital was based on its comprehensive coverage of medical departments, which aligns well with the study's focus on the application of doctor-patient trust in the management of multiple diseases. Additionally, the hospital has a high annual patient volume, ensuring sufficient data saturation and sample representativeness. The research team has maintained a long-term collaboration with the hospital, ensuring the compliance and efficiency of data acquisition.

According to the proportional questionnaire calculation formula, with a 95% confidence level, 5% margin of error, and an expected proportion of *p* = 0.5, at least 384 valid samples are required. The study collected questionnaire data from a tertiary hospital in Jiangxi Province between June 1 and December 31, 2024, covering outpatients from all departments. A total of 834 valid responses were obtained, of which 672 were valid, with a validity rate of 80.58%. No major adjustments were made to medical policies during the study period, thereby minimizing interference from external variables. The subjects were individuals who had visited the hospital or accompanied family members to the hospital. All participants provided informed consent.

### 2.2. Measures

#### 2.2.1. Physician empathy scale

The Consultation and Relational Empathy Measure (CARE) was developed in 2004 by Professor Stewart W. Mercer of the University of Glasgow and the University of Edinburgh in the UK to quantify the degree of physician empathy perceived by patients at the conclusion of a consultation. The scale comprises a total of 10 items, which are divided into 4 dimensions: Dimension 1: Communication (CARE items 1, 2, and 3), Dimension 2: Overall Assessment (CARE item 4), Dimension 3: Understanding and Compassionate Response (CARE items 5 and 6), and Dimension 4: Empowerment (CARE items 7–10). Higher scores indicate a greater degree of empathy on the part of the physician (13).

#### 2.2.2. Patient trust

The Patient Trust Scale, a tool developed by Xiaokang Lu, was utilized in this study. This scale was further subdivided into a presuppositional subscale and a realistic subscale. Scoring on this scale is conducted on a 5-point scale, with higher scores denoting greater agreement. In this study, we examined patients' specific perceptions of their doctors and hospitals during the course of medical treatment, utilizing the realism trust subscale of the Patient Trust Scale. The homogeneity reliability coefficients (Cronbach's α) of the two subscales of the patient's trust scale are above 0.7. The 2-week retest reliability of the patient's realism subscale is 0.69, the split-half reliability is 0.85, and the Kendall's harmony coefficient is 0.81 (14).

#### 2.2.3. Patient satisfaction

The Chinese PDRQ-15 scale, translated and revised by Hui (15), was selected for this study. This scale consists of 15 items, which are divided into three dimensions: patients' satisfaction with doctors, doctors' approachability, and patients' attitudes toward medical symptoms. Each dimension has 6, 7, and 2 items, respectively. The scale is structured on a five-point Likert scale, with 1 indicating very non-compliant, 2 indicating non-compliant, 3 indicating fair/unsure, 4 indicating compliant, and 5 indicating very compliant, with higher scores denoting higher patient satisfaction. Six questions were measured using one of the dimensions of patient satisfaction with physicians, which were “1. My physician knows me.”, “2. I trust my physician.”, “3. My physician is conscientious about helping me.”, “4. I can talk freely with my physician.”, “5. I am satisfied with my practitioner's treatment.”, and “6. I feel better thanks to my physician” (15).

#### 2.2.4. Hypothesis

*H1:* Physician empathy exerts a substantial influence on patient trust, with increased physician empathy corresponding to heightened patient trust.
*H2:* Patient satisfaction serves as a mediating factor in the relationship between physician empathy and patient trust. Physician empathy has been demonstrated to be an effective strategy for increasing patient satisfaction, and increased patient satisfaction has been shown to further promote patient trust in physicians.

### 2.3. Ethical considerations

The present study was conducted in accordance with national and international standards for the protection of research subjects, including the Declaration of Helsinki and the Ethical Review Measures for Life Science and Medical Research Involving Human Subjects. The present study employed the use of anonymous questionnaires, ensuring the complete confidentiality of the respondents' responses. The collection of biological samples or sensitive information was not a component of the study, thus rendering written informed consent unnecessary. It is imperative to note that the maintenance of confidentiality for these records is of the utmost importance. In the event of publication of the research results, the identity information of the participants will not be disclosed.

### 2.4. Results

#### 2.4.1. Demographic variables

This study used SPSS 23.0 for data analysis.

The demographic variables were analyzed using descriptive statistics. These demographic variables were then used as control variables in independent samples *t*-tests and one-way ANOVAs. The age of the subjects was categorized into three stages: young, middle-aged, and old. The sample included 252 males (37.5%) and 420 females (62.5%). The age distribution was as follows: 406 (60.4%) subjects were between 18 and 40 years of age, 204 (30.4%) subjects were between 41 and 65 years of age, and 62 (9.2%) subjects were over 66 years of age. The geographical distribution of the subjects was as follows: 334 (49.7%) resided in urban areas, while 338 (50.3%) resided in rural areas. Furthermore, the educational attainment of the participants was as follows: 284 (42.3%) were in high school or below, 105 (15.6%) were in college, 258 (38.4%) were in undergraduate studies, and 25 (3.7%) were in graduate school or above.

The findings indicated that gender (*p* = 0.048), age (*p* = 0.000), education (*p* = 0.000), and the department of last visit (*p* = 0.003) significantly influence patient trust, place of birth does not significantly affect patient trust (*p* = 0.640). Consequently, in subsequent analyses, gender, age, education, and department were incorporated as control variables as covariates for mediation effect analysis to eliminate the effect of control variables on the dependent variable.

#### 2.4.2. Common method bias

In order to control the interference of systematic measurement errors on research conclusions, the common method bias of the questionnaire was examined using the Harman one-way test. The results showed that six factors had an Eigen root >1 and the first factor had an explanatory rate of 36.35%, which was below the critical criterion of 40%, indicating that the data in this study did not suffer from serious common method bias.

#### 2.4.3. Descriptive statistics and correlation analysis

Before conducting a mediation analysis, first ensure that there is a correlation between the variables. The variables were correlated and the results are shown in Table 1. There was a significant positive correlation between physician empathy, patient satisfaction and patient trust.

**Table 1:** Mean, standard deviation and correlation coefficients for each study variable.

**Variant**	***M***	***SD***	**1**	**2**	**3**
1. Physician empathy	8.69	2.802	1		
2. Patient satisfaction	23.08	5.267	0.389^**^	1	
3. Patient trust	49.40	9.119	0.462^**^	0.590^**^	1

^**^p < 0.01.

#### 2.4.4. Regression analysis and mediation effects

In order to verify the relationship between independent samples on patient trust, regression analysis was used, and in order to verify the problem of multicollinearity, the values of tolerance and VIF were examined, and in general, the value of VIF is < 10, and the tolerance is >0.1, and there is no problem of multicollinearity. Through systematic multicollinearity diagnosis, it can be ensured that the research conclusions satisfy both statistical rigor and domain explanatory power. As shown in Table 2, the results indicated that physician empathy significantly and positively predicted patient trust (β = 0.274, *p* < 0.001) and patient satisfaction significantly and positively predicted patient trust (β = 0.483, *p* < 0.001).

**Table 2:** Regression analysis of independent variables on patient trust.

**Mold**		**Unstandardized coefficient**		**Standardized coefficient**	***t***	***p***	**Covariance statistics**	
		***B***	**Standard error**	**Beta**			**Tolerances**	**VIF**
1	(constant)	22.345	1.283		17.423	0.000		
	Physician empathy	0.892	0.105	0.274	8.518	0.000	0.849	1.178
	Patient satisfaction	0.837	0.056	0.483	15.012	0.000	0.849	1.178

R = 0.642, R^2^ = 0.412, adjusted R^2^ = 0.410, F = 234.085.

**Figure 1:**
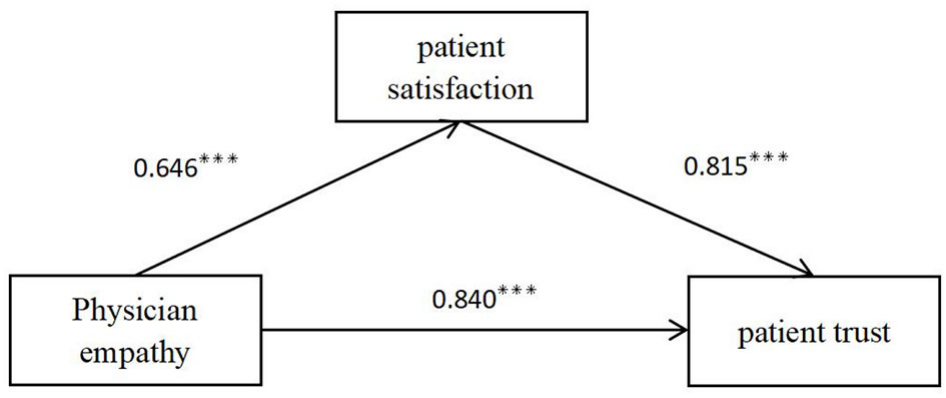
Intermediary model. ***p < 0.001.

The mediated effects analysis was performed using spss23.0 and PROCESS v3.5 plug-in. Before the model test, the data were standardized, and the samples were sampled 5,000 times repeatedly using the Bootstrap method, and 95% confidence intervals were calculated to further test the significance of the mediating effect. The results are shown in Table 3 and Figure 1. The results of the mediation analysis showed that the direct effect value was 0.840, with a 95% CI of [0.629, 1.051], which did not include 0, indicating that physician empathy directly and positively affects patient trust. The mediating effect value was 0.526, with a 95% CI of [0.070, 0.397], not including 0. This indicates that the mediating effect of patient satisfaction is significant in the effect of physician empathy on patient trust, suggesting that patient satisfaction plays a partially mediating role in the middle of the physician empathy where patient trust is concerned.

**Table 3:** Results of the mediation effect test.

**Effect**	**Trail**	**Effect value**	**Boot standard error**	**95%CI**	
				**LL**	**UL**
Direct effect	Physician empathy → patient trust	0.840	0.107	0.629	1.051
Intermediary effect	Physician empathy → patient satisfaction → patient trust	0.526	0.070	0.397	0.672
Total effect		1.367	0.116	1.139	1.594

## 3 Discussion

This study is based on the following theoretical framework. The theoretical framework of social exchange emerged in the late 1950s, drawing from the tenets of utilitarian economics and psychological behaviorism. This theory gained widespread traction within the social sciences, particularly in fields such as sociology and psychology. The theory posits that individuals with different levels of resources engage in exchanges with each other to fulfill their personal needs (16). According to the social exchange theory, the demonstration of empathy by a physician is associated with patients' perceptions of the physician's care and support, which in turn fosters greater patient trust and the development of a positive relationship with the physician. Conversely, the affective trust theory posits that a physician's empathy fosters heightened affective trust in the patient, leading to the belief that the physician will provide high-quality medical care that aligns with the patient's best interests.

The mediating role of patient satisfaction between physician empathy and patient trust can be explained from the perspective of expectation confirmation theory. Initially applied primarily in the traditional business field, Expectancy Confirmation Theory posits that discrepancies between consumers' initial expectations and actual experiences give rise to varying levels of confirmation. This variability in confirmation levels, in turn, impacts purchase intention through satisfaction as a mediator (17). Within the context of the doctor-patient relationship, when a physician's level of empathy exceeds the patient's expectations, the patient experiences satisfaction with the physician's service, thereby fostering increased trust in the physician (17).

This study examined the effect of physician empathy on patient trust and tested the mediating role of patient satisfaction in this relationship. The findings support the hypothesis that higher levels of physician empathy are associated with higher levels of patient trust, and that patient satisfaction plays a partial mediating role in this relationship. In this study, we examined the impact of physician empathy on patient trust, and the findings further highlight the critical importance of establishing and maintaining the “golden triangle” of physician empathy, patient satisfaction, and patient trust in enhancing the quality of healthcare services.

From the patient's perspective, the robust establishment of the “golden triangle” has the potential to significantly improve the patient's healthcare experience. Empathy, defined as the ability to understand and share another person's feelings, is a fundamental component of medical professionalism. Research has demonstrated that patients who experience high levels of empathy from their healthcare providers tend to feel respected and understood, thereby fostering a stronger sense of trust in the physician-patient relationship. Trust in the healthcare provider is a fundamental prerequisite for active cooperation in treatment. Patients who have confidence in their physicians are more likely to adhere to medical directives, adhere to medication schedules, undergo diagnostic procedures, and participate in rehabilitation exercises. This, in turn, enhances treatment adherence, improves treatment outcomes, and increases patient satisfaction. For hospitals, the effective implementation of the “golden triangle” has been demonstrated to contribute to a reduction in the occurrence of medical disputes. In the event of unfavorable outcomes, patients demonstrate a propensity to engage in communication and negotiation with medical professionals, thereby facilitating the seamless delivery of healthcare services.

### 3.1. The impact of physician empathy on patient trust

The results of this study showed a 95% CI for the direct effect of [0.629, 1.051], indicating that physician empathy directly and positively affects patient trust. This is consistent with several studies in recent years, confirming the positive impact of physician empathy on patient trust (H1).The manifestation of physician empathy is evident in the understanding of patients' emotions, the demonstration of empathic ability, and the expression of emotional support. These behaviors have the potential to significantly enhance patients' trust in their physicians. Empathy is a pivotal factor in effective communication between physicians and patients and is regarded as an essential element of the doctor-patient relationship (18). Empathy has been demonstrated to improve patients' trust in medical staff, satisfaction, and influence treatment adherence as well as treatment outcomes (10). Derksen demonstrated that physician empathy significantly enhances patients' trust in physicians, particularly in the context of chronic disease management (19). This trusting relationship has a substantial impact on patients' treatment adherence and prognosis. Additionally, Neumann et al. (9) found that physician empathy not only enhances patient trust but also reduces patient anxiety and depression, thereby improving the overall patient experience.

In light of the evolving healthcare paradigm, patient expectations of physicians have expanded beyond technical competence to encompass emotional support and humanistic care. Empirical evidence has demonstrated that physician empathy has the capacity to substantially augment patient trust, particularly within the context of strained physician-patient relationships (20). During the outbreak of the novel coronavirus, for instance, physicians' demonstration of empathy was particularly important in building patient trust. Studies have demonstrated that patients' trust in physicians exhibits a marked increase during outbreaks, a phenomenon that is closely associated with physicians' high levels of empathy and responsibility during crises (21).

### 3.2. The mediating role of patient satisfaction

In this study, patient satisfaction was introduced as a mediating variable into the relationship model between physician empathy and patient trust, and the results showed that the 95% CI of the mediating effect was [0.070, 0.397], which indicated that the mediating effect of patient satisfaction was significant in the influence of physician empathy on patient trust, suggesting that patient satisfaction partially mediated the relationship between physician empathy and patient trust, and verified its partial mediating role (H2).This finding suggests that physician empathy can directly affect patient trust and, in turn, enhance patient trust by improving patient satisfaction. In the context of contemporary medical services, it is imperative to adopt a patient-centered approach, attentive to patients' emotional needs and cognizant of their social attributes (22). Patient satisfaction has been identified as a critical component in the assessment of healthcare service quality. This term encompasses not only patients' immediate perceptions of service quality but also serves as a pivotal factor influencing patient behaviors and attitudes. In recent years, numerous studies have examined the impact of patient satisfaction on the doctor-patient relationship. The extant literature suggests that patient satisfaction has a significant impact on patients' trust in their doctors, with this effect being consistent across different types of healthcare services (11).

The patient-centered medical model has gained significant popularity in recent years, and with it, the importance of patient satisfaction has become increasingly evident. Research has demonstrated that patient satisfaction not only fosters patients' trust in physicians but also enhances treatment adherence and overall health (22). Consequently, healthcare organizations must prioritize the enhancement of patient satisfaction by improving the patient experience through enhancing the empathy of physicians.

### 3.3. Contributions and innovations

For primary care physicians, patient trust is the cornerstone of a good doctor-patient relationship. Empathy, defined as the ability to understand and share the feelings of another individual, is a crucial component of primary care. Research has demonstrated that when primary care physicians exhibit high levels of empathy, patients develop a greater sense of trust in them. This increased trust fosters patients' willingness to adhere to the physician's recommendations and treatment plans, leading to enhanced treatment compliance. Empathy is a crucial component of primary care, enabling physicians to comprehend patients' needs, concerns, and expectations. This ability facilitates the effective communication of medical information, enhancing its acceptance and comprehension among patients. Primary care physicians are charged with the responsibility of addressing not only their patients' physical health but also their mental wellbeing. Empathy is a crucial component of primary care, enabling physicians to discern subtle changes in patients' emotional states and psychological distress. This ability facilitates the provision of timely emotional support and comfort, fostering a sense of wellbeing and facilitating recovery.

The findings of this study indicate that medical institutions should prioritize the cultivation of doctors' empathy. One strategy to achieve this objective is to implement empathy training and incorporate empathy assessment into the performance evaluation system for doctors. This approach is expected to enhance the level of doctors' empathy, thereby improving patient satisfaction and trust, and fostering a harmonious doctor-patient relationship.

Specifically, medical institutions can implement the following measures. Firstly, medical institutions can implement regular empathy training to assist doctors in acquiring fundamental empathy skills, including active listening, emotional response, and empathic expression. Through simulation training, role-playing, and other methods, doctors can enhance their empathy skills in practice. Secondly, medical institutions can incorporate empathy into performance appraisal. They can assess doctors' empathy performance using patients' feedback, peers' evaluations, and other methods. The assessment results can be linked to doctors' promotion and salary, among other factors. This approach incentivizes doctors to enhance their empathy level. Thirdly, medical institutions can optimize the mode of doctor-patient communication. They can encourage doctors to prioritize patients' emotional and psychological needs during diagnosis and treatment, fostering effective communication and interaction to enhance patients' trust and satisfaction.

In the prevailing training and evaluation system for healthcare professionals, effective doctor-patient communication is a requisite skill and a cost-effective intervention for the doctor-patient relationship. Research has indicated that effective communication between doctors and patients plays a pivotal role in mitigating tensions within the doctor-patient relationship, promoting patient value recognition, and enhancing patient satisfaction (23, 24).The establishment of patient feedback mechanisms is a crucial aspect of healthcare organizations. By implementing regular patient feedback mechanisms, healthcare organizations can collect patient evaluations of doctor empathy and satisfaction, and utilize the feedback to continuously enhance and optimize the quality of medical services. A focus on the outpatient experience necessitates that healthcare organizations understand the concerns of all types of patients. It is imperative that healthcare organizations encourage patients to voice their concerns in the first place and address them. This encouragement is a driving force for improving healthcare services (25).

### 3.4. Shortcomings and prospects

Notwithstanding the valuable findings of this study, there are some limitations that must be acknowledged. Firstly, the present study employed a cross-sectional design; it would be advantageous for future studies to utilize a longitudinal or experimental design to further validate the findings of this study. For instance, the long-term implications of physician empathy on patient trust and satisfaction could be investigated through the implementation of follow-up surveys or intervention experiments. Secondly, future studies could utilize multiple sources of data. For instance, the level of physician empathy could be assessed through physician self-assessment scales or third-party observation records, thereby enhancing the objectivity and accuracy of the data. Finally, the current study's sample size was constrained to outpatients from a single hospital, which limits the generalizability of the findings. Future studies should aim to expand the sample size and scope to enhance the external validity of the results. To enhance the applicability of the study's findings to diverse healthcare settings and patient populations, multicenter studies or large-scale surveys could be conducted. The exploration of additional mediating or moderating variables, such as patient personality traits and doctor-patient communication patterns, could offer a more comprehensive understanding of the mechanisms through which physician empathy affects patient trust.

## 4 Conclusion

In summary, the present study revealed the positive impact of physician empathy on patient trust and verified the partial mediating role of patient satisfaction in this relationship. This finding not only enriches the research on the mechanism of the role of physician empathy, but also provides new ideas for healthcare organizations to enhance patient trust. Consequently, medical institutions must prioritize the cultivation of doctors' empathy, enhancing it through empathy training and integrating empathy into performance evaluation. This approach will lead to improved patient satisfaction and trust, thereby fostering a harmonious doctor-patient relationship.

## Data Availability

The raw data supporting the conclusions of this article will be made available by the authors, without undue reservation.
